# Association of retinal neurodegeneration with the progression of cognitive decline in Parkinson’s disease

**DOI:** 10.1038/s41531-024-00637-x

**Published:** 2024-01-23

**Authors:** Ane Murueta-Goyena, David Romero-Bascones, Sara Teijeira-Portas, J. Aritz Urcola, Javier Ruiz-Martínez, Rocío Del Pino, Marian Acera, Axel Petzold, Siegfried Karl Wagner, Pearse Andrew Keane, Unai Ayala, Maitane Barrenechea, Beatriz Tijero, Juan Carlos Gómez Esteban, Iñigo Gabilondo

**Affiliations:** 1 Neurodegenerative Diseases Group, Biobizkaia Health Research Institute, Barakaldo, Spain; 2 Department of Neurosciences, Faculty of Medicine and Nursery, University of the Basque Country (UPV/EHU), Leioa, Spain; 3 Biomedical Engineering Department, Faculty of Engineering (MU-ENG), Mondragon Unibertsitatea, Mondragón, Spain; 4 NIHR Biomedical Research Centre at Moorfields Eye Hospital and UCL Institute of Ophthalmology, EC1V 2PD London, UK; 5 Department of Ophthalmology, Araba University Hospital, Vitoria-Gasteiz, Spain; 6 Department of Neurology, Donostia University Hospital, Donostia, Spain; 7 Biogipuzkoa Health Research Institute, Donostia, Spain; 8 CIBERNED, Institute of Health Carlos III, Madrid, Spain; 9 Queen Square Institute of Neurology, University College London, London, UK; 10 The National Hospital for Neurology and Neurosurgery, London, UK; 11 Departments of Neurology and Ophthalmology, Amsterdam UMC, Amsterdam, Netherlands; 12 Institute of Ophthalmology, University College London, London, UK; 13 Neurology Department, Cruces University Hospital, Barakaldo, Spain; 14 IKERBASQUE, The Basque Foundation for Science, Bilbao, Spain

**Keywords:** Parkinson's disease, Prognostic markers, Neurodegeneration, Visual system, Parkinson's disease

## Abstract

Retinal thickness may serve as a biomarker in Parkinson’s disease (PD). In this prospective longitudinal study, we aimed to determine if PD patients present accelerated thinning rate in the parafoveal ganglion cell-inner plexiform layer (pfGCIPL) and peripapillary retinal nerve fiber layer (pRNFL) compared to controls. Additionally, we evaluated the relationship between retinal neurodegeneration and clinical progression in PD. A cohort of 156 PD patients and 72 controls underwent retinal optical coherence tomography, visual, and cognitive assessments between February 2015 and December 2021 in two Spanish tertiary hospitals. The pfGCIPL thinning rate was twice as high in PD (*β* [SE] = −0.58 [0.06]) than in controls (*β* [SE] = −0.29 [0.06], p < 0.001). In PD, the progression pattern of pfGCIPL atrophy depended on baseline thickness, with slower thinning rates observed in PD patients with pfGCIPL below 89.8 µm. This result was validated with an external dataset from Moorfields Eye Hospital NHS Foundation Trust (AlzEye study). Slow pfGCIPL progressors, characterized by older at baseline, longer disease duration, and worse cognitive and disease stage scores, showed a threefold increase in the rate of cognitive decline (*β* [SE] = −0.45 [0.19] points/year, *p* = 0.021) compared to faster progressors. Furthermore, temporal sector pRNFL thinning was accelerated in PD (*β*_time x group_ [SE] = −0.67 [0.26] μm/year, *p* = 0.009), demonstrating a close association with cognitive score changes (*β* [SE] = 0.11 [0.05], *p* = 0.052). This study suggests that a slower pattern of pfGCIPL tissue loss in PD is linked to more rapid cognitive decline, whereas changes in temporal pRNFL could track cognitive deterioration.

## Introduction

Optical coherence tomography (OCT) has emerged as a valuable tool for assessing retinal changes associated with neurodegenerative diseases, including Parkinson’s disease (PD). OCT enables high-resolution, reproducible, and precise measurements of retinal layer thicknesses for detecting structural alterations. Among the retinal layers, the ganglion cell-inner plexiform layer (GCIPL) has garnered substantial attention due to its potential as a biomarker for neurodegeneration and cognitive decline^1–5^.

Identifying PD patients at risk of cognitive decline poses a significant challenge for patient stratification in clinical trials and effective clinical management. Several studies have now provided evidence that visual disability can predict cognitive impairment and dementia in PD^2,4,6,7^. Our previous study demonstrated that OCT measures can reliably identify PD patients with and without visual impairment, primarily by assessing parafoveal GCIPL (pfGCIPL) thickness^1^. Subsequently, we observed that a reduced pfGCIPL thickness at a single time point could predict the risk of global cognitive decline^2^. Similar observations have also been identified in the peripapillary retinal nerve fiber layer (pRNFL). According to Zhang et al., low pRNFL thickness was associated with greater annualised decline in global cognition over a 3-year period^8^. However, the wide range of retinal thickness distribution in the normal population^9–12^ and the lack of agreement between different OCT technologies and devices^13–15^ make it challenging to establish a universal cut-off value for identifying PD patients with “low” retinal thickness. Instead, using rates of retinal thinning overcomes the challenges of relying solely on single thickness values, thereby enhancing the generalisability and applicability of OCT into clinical settings for predicting clinical outcomes.

In a previous study, we reported that the rate of pfGCIPL thinning in PD was significantly higher compared to controls but confirmatory studies are still needed^2^. Recent evidence suggests that pfGCIPL thinning and cognitive deterioration do not progress in parallel^6^, indicating the existence of complex dynamics between retinal neurodegeneration and cognition. On the other hand, longitudinal studies tracking the evolution of pRNFL thickness in PD and its correlation with clinical progression are still lacking. Understanding the temporal relationship between retinal changes and cognitive impairment in PD is critical for exploring the retina as a potential biomarker for the disease monitoring.

Therefore, the objectives of this work are twofold. Firstly, we intend to confirm that the rate of pfGCIPL thinning is higher in PD than in controls by testing our hypothesis in an extended sample of participants and by validating the results in an external OCT database. Additionally, we seek to explore whether pRNFL follows a similar dynamic. Secondly, we aim to investigate the relationship between the thinning rates of pfGCIPL and pRNFL and the progression of clinical scores in PD.

## Results

### Demographic and clinical characteristics of study participants

The baseline demographic and clinical characteristics of study participants are summarized in Table 1. The mean [SD] age of the control group was 61.4 [7.5] years, which was significantly lower than the PD group with a mean [SD] age of 64.8 [8.7] years (*p* = 0.002). The proportion of females was significantly higher in the control group, and the mean [SD] disease duration was 6.02 [4.7] years.

**Table 1:** Descriptive statistics of baseline outcomes.

	Control		PD			
	*n*	Mean (SD)	*n*	Mean (SD)	*p* value	
Demographics						
Age (years old)	72	61.4 (7.5)	156	64.8 (8.7)	0.002	
Sex, *n* (% females)	72	41 (57.7)	156	55 (34.8)	0.002	
Education years	72	12.2 (3.4)	156	11.0 (3.6)	0.025	
White race, no. (%)	72	72 (100)	156	156 (100)	−	
Disease-related variables						
Disease duration (years)	–	NA	156	6.02 (4.7)	NA	
Age at disease onset (years)	–	NA	156	58.8 (8.5)	NA	
H&Y stage	–	NA	156	2 [2–2.5]	NA	
UPDRS I	–	NA	153	2.0 (1.7)	NA	
UPDR S II	–	NA	153	9.7 (5.7)	NA	
UPDRS III	–	NA	154	23.9 (11.3)	NA	
UPDRS IV	–	NA	153	3.3 (3.3)	NA	
LEDD (mg)	–	NA	156	607.6 (353.2)	NA	

	*n*	Mean(SD)	*n*	Mean (SD)	*β* (SE)	Adjusted *p*
Cognitive outcomes						
MoCA^a^	71	26.1 (2.4)	154	23.3 (4.5)	−2.40 (0.55)	<0.001
Benton Line Orientation Judgment	65	23.1 (4.1)	122	19.8 (5.9)	−2.6 (0.70)	<0.001
Salthouse Perceptual Comparison Test	67	24.7 (8.4)	124	20.6 (8.5)	−1.92 (0.98)	0.048
Symbol Digit Modality Test	67	45.6 (10.8)	122	31.7 (13.2)	−9.8 (1.6)	<0.001
Trail Making Test, part-A ^b^	67	38.5 (11.7)	124	53.9 (26.6)	11.9 (3.1)	<0.001
Trail Making Test, part-B ^b^	57	89.0 (32.0)	116	149.7 (85.2)	50.7 (10.4)	<0.001
Modified Wisconsin Card Sorting Test	56	5.2 (1.7)	93	4.0 (2.1)	−1.0 (0.30)	<0.001
Primary visual outcomes						
High Contrast Visual Acuity ^c^	35	63 (4.4)	72	60.1 (5.4)	−2.30 (0.97)	0.018
High Contrast Visual Acuity^d^	36	44.3 (3.8)	77	42.6 (4.7)	−1.16 (0.83)	0.165
Low Contrast Visual Acuity^c^	34	38.9 (6.7)	72	28.5 (11.0)	−8.63 (1.95)	<0.001
Low Contrast Visual Acuity^d^	36	29.4 (6.5)	77	22.7 (10.0)	−5.24 (1.7)	0.003
Contrast Sensitivity, photopic^e^	34	2.05 (0.14)	72	1.91 (0.14)	−0.11 (0.03)	<0.001
Contrast Sensitivity, mesopic^e^	34	1.77 (0.1)	72	1.66 (0.14)	−0.10 (0.02)	<0.001
Contrast Sensitivity, contrast %^b,d^	34	1.09 (0.29)	77	1.83 (1.23)	0.53 (0.20)	0.007
Contrast Sensitivity, number of letters^d^	34	73.97 (1.88)	77	65.6 (16.8)	−8.1 (2.1)	<0.001

The estimated differences between groups were calculated with LMMs adjusted for age at baseline, sex and education years for cognitive scores, and adjusted for age at baseline and sex for primary visual outcomes. *β* indicates the adjusted difference between groups. PD group was used as the reference group.

*LEDD* levodopa-equivalent daily dose, *H&Y* Hoenh&Yahr scale, *MoCA* Montreal Cognitive Assessment, *NA* not applicable.

^a^ Global cognition was assessed with MoCA, a validated instrument whose scoring system ranges from 0 to 30. Scores below 26 are indicative of mild cognitive impairment.

^b^ Higher scores indicate worse performance (i.e., more seconds to complete the task or more contrast needed to identify letters).

Due to a protocol change, primary visual function was measured differently:

^c^ Retro-illuminated cabinet at 4 meter using 100% contrast ETDRS and 2.5% contrast Sloan charts.

^d^ Precision Vision Visual Acuity Test (PVVAT) digital software at 4 m.

^e^ Pelli-Robson Test at 1 m.

At baseline, the PD group had lower scores in all cognitive tests, lower contrast visual acuity and contrast sensitivity compared to the control group after adjustment (Table 1). About 68% of participants had at least one follow-up visit with a mean [SD] follow-up time of 2.7 [1.7] years (ranging from 1 to 5 years) and 2.3 [0.45] follow-up visits (Supplementary Fig. 1). There was a significant cognitive decline (MoCA) in PD patients (*β* [SE] = −0.22 [0.09], *p* = 0.023) but not in controls (*β* [SE] = 0.04 [0.10], *p* = 0.675). Among the remaining cognitive and visual variables, the majority did not exhibit significantly different rates between PD and controls (Supplementary Table 1), except for Salthouse Perceptual Comparison Test and high-contrast visual acuity measured with digital software, which progressed faster in PD patients.

In the OCT validation dataset, the proportion of males was similar between groups (60.6% controls and 55.7% PD). On average, PD patients (76.7 [8.6] years old) and controls (75.7 [9.4] years old) were older than in test dataset (Supplementary Table 2).

### Accelerated pfGCIPL thinning rate in PD

**Fig. 1:**
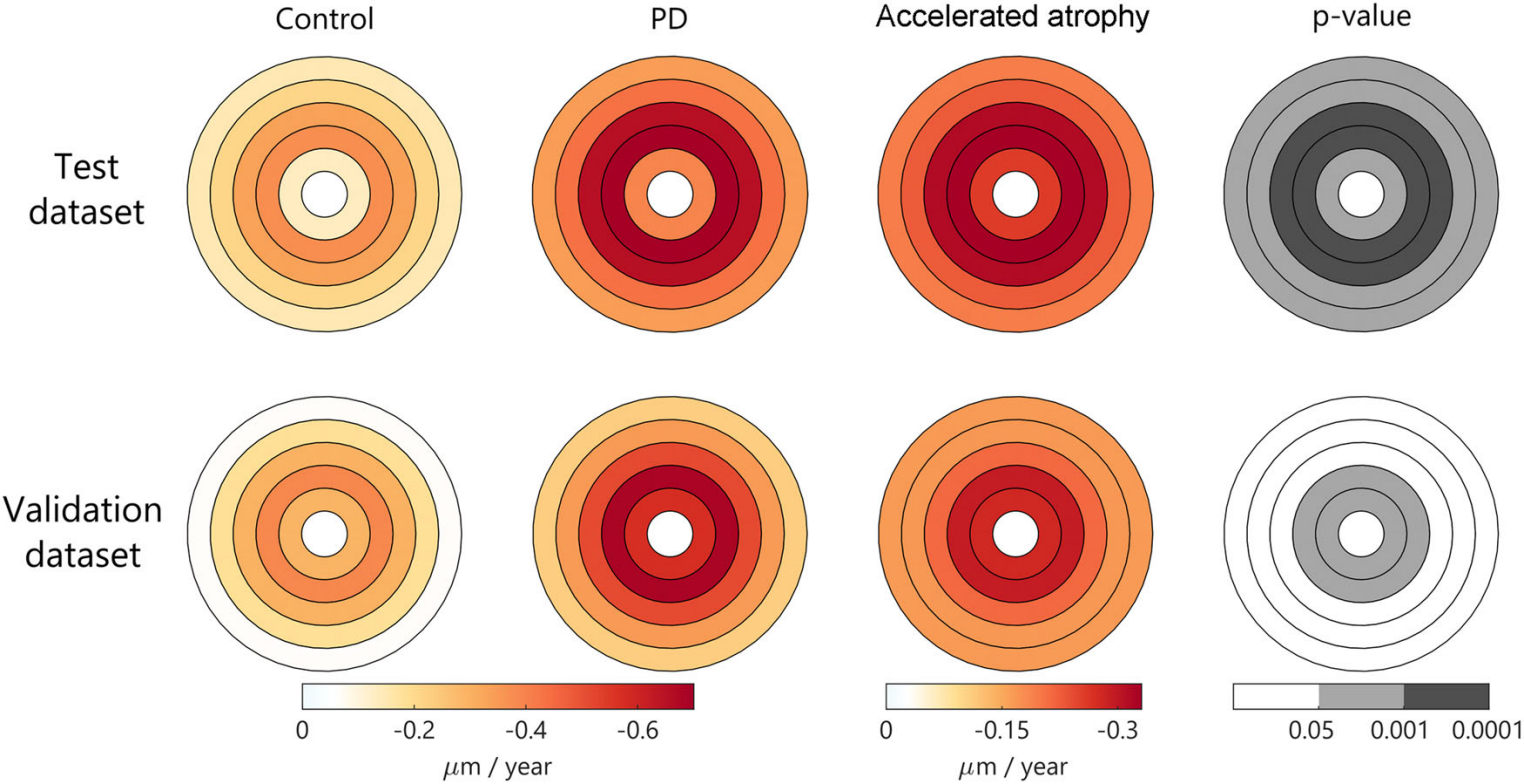
Annualised estimates of GCIPL thinning rate. Using linear mixed-effects models adjusted for age at baseline and sex. Color represents the estimated atrophy rate in each foveo-centered area. Absolute rates are represented in the first two columns. The relative increase in PD vs. control is represented on the third column, and the corresponding significant p-values for group effect are represented in gray scale. *Abbreviations:* GCIPL: ganglion cell-inner plexiform layers; PD, Parkinson’s disease.

In the test dataset, the group effect at baseline was not significant for pfGCIPL thickness (group PD, *β* [SE] = 1.28 [1.11], *p* = 0.251), but pfGCIPL consistently correlated with disease-related outcomes, showing a significant correlation with global cognition, H&Y stage, and disease duration at baseline (Supplementary Fig. 2). A longitudinal analysis of retinal thickness in foveo-centered concentric annuli revealed that the thinning rate in PD patients was higher compared to controls. From all macular layers and circular sectors, GCIPL thickness in the parafoveal regions showed the largest estimates of annualised changes and the largest differences between PD and controls (Fig. 1). The estimated annual change of pfGCIPL was twice as high in PD patients as in controls (Table 2), being this difference statistically significant (*β* [SE] _time *x* group_ = −0.29 [0.08], *p* < 0.001). The GCIPL thinning was also significantly increased in more peripheral rings of PD patients compared to controls but the thinning rates were slower than for pfGCIPL.

**Table 2:** Estimated annualised pfGCIPL thinning rate.

		*ß*_time_ (SE)	*p* value	*ß*_interaction_ (SE)	*p* value
Test dataset	n				
PD	156	−0.58 (0.06)	<0.001	−0.29 (0.08)	<0.001
Control	72	−0.29 (0.06)	<0.001		
PD, high pfGCIPL	90	−0.65 (0.07)	<0.001	0.25 (0.12)	0.042
PD, low pfGCIPL	66	−0.39 (0.10)	<0.001		
Control, high pfGCIPL	45	−0.29 (0.07)	<0.001	0.004 (0.14)	0.977
Control, low pfGCIPL	27	−0.28 (0.12)	0.029		
Validation dataset					
PD	167	−0.66 (0.08)	<0.001	−0.27 (0.10)	0.008
Control	873	−0.38 (0.04)	<0.001		
PD, high pfGCIPL	115	−0.73 (0.10)	<0.001	0.32 (0.18)	0.110
PD, low pfGCIPL	52	−0.40 (0.16)	0.120		
Control, high pfGCIPL	655	−0.38 (0.05)	<0.001	−0.05 (0.12)	0.646
Control, low pfGCIPL	218	−0.41 (0.10)	<0.001		

*ß*_time_ represents the estimated coefficient of the annualised pfGCIPL thinning rate (μm/year) derived from age- and sex-adjusted LMMs. *ß*_interaction_ indicates the increased thinning rate in PD or high pfGCIPL subgroup compared to controls or low pfGCIPL subgroup, respectively.

*pfGCIPL* parafoveal ganglion cell-inner plexiform layers, *SE* standard error.

To validate the current findings, OCTs from validation dataset were analyzed. The results were consistent with our observations. First, we found an increased pfGCIPL thinning rate in PD patients compared to controls (*β* [SE] _time *x* group_ = −0.27 [0.10], *p* = 0.008; Fig. 1). Indeed, the rate of pfGCIPL thinning was similar to that observed in our cohort (Table 2). pfGCIPL was the only macular area that thinned at a faster rate in PD than in controls, as the remaining annuli did not show statistically significant differences between groups.

### Different patterns of pfGCIPL thinning rate within PD

In view of the accelerated pfGCIPL atrophy rate in PD, we next tested whether the retinal atrophy rate was homogeneous across subjects. First, in an exploratory analysis, we selected PD individuals who had OCT data available for three time points. These analyses revealed that the model with a correlated random slope and intercept had a significantly better fit (Akaike Information Criterion, [AIC] = 635.1) than the model with uncorrelated random slope and intercept (AIC = 814.4), suggesting that baseline pfGCIPL thickness was significantly associated with the rate of subsequent pfGCIPL thinning. Therefore, we divided participants into two subgroups based on their initial pfGCIPL thickness as an approach to explore the non-constant rate of pfGCIPL atrophy within all included PD subjects, because the number of observations per participant did not allow fitting random slopes in the whole dataset. Table 2 shows the estimated annualised changes of pfGCIPL in each subgroup. PD patients with low baseline pfGCIPL thickness presented 40% to 45% slower rates of retinal atrophy over time, in the test and validation datasets, respectively. Further adjusting the models for disease duration at baseline did not lead to different outcomes (Supplementary Table 3). No differences were observed in controls according to the pfGCIPL subgroup (Supplementary Table 4).

### Baseline differences according to pfGCIPL atrophy rate pattern

There were significant differences in age, sex, disease duration, and age at disease onset between PD patients depending on their baseline pfGCIPL thickness (Table 3). Specifically, low pfGCIPL PD patients - who showed slow pfGCIPL progression pattern - were significantly older, had a higher proportion of female patients, and presented longer disease duration. In this group, male and female PD patients demonstrated similar age, disease duration, and disease stage severity. No differences were observed in LEDD or years of education between pfGCIPL atrophy rate patterns. Similarly, in the validation cohort PD patients with low pfGCIPL were, on average, 4 years older (*p* = 0.003; Supplementary Table 5). Furthermore, in PD patients with slow progressing pfGCIPL pattern, baseline global cognition was significantly worse after adjusting for the age at entry, sex, and years of education, whereas baseline H&Y stage was higher (Supplementary Table 6). They also showed worse baseline cognitive scores in tests assessing visual attention and processing speed and visual executive functions, whereas primary visual function was comparable among fast and slow pfGCIPL progressing PD subgroups (Table 3 and Supplementary Table 6). Both PD subgroups presented lower cognitive and visual scores than control participants. In controls, we did not observe statistically significant differences in baseline cognitive scores according to pfGCIPL subgroup (Supplementary Table 4). Finally, PD patients with slow progressing pfGCIPL pattern not only exhibited lower macular thickness but also reduced pRNFL thickness across all its sectors at baseline (Supplementary Table 7).

**Table 3:** Differences in demographics and clinical characteristics between high and low pfGCIPL subgroups in PD.

	Control		PD, High pfGCIPL		PD, Low pfGCIPL		*p*-value^a^	*p*-value^b^
	*n*	Mean (SD)	*n*	Mean (SD)	*n*	Mean (SD)		
Demographics								
Age (years old)	72	61.4 (7.5)	90	62.9 (8.2)	66	68.6 (7.9)	0.614	<0.001***
Sex, *n* (% females)	72	41 (57.7)	90	24 (26.7)	66	31 (47.0)	<0.001	0.014*
Education years	72	12.2 (3.4)	90	12.2 (3.5)	66	12.3 (3.2)	0.195	0.855
White race, no. (%)	72	72 (100)	90	90 (100)	66	66 (100)	NA	NA
Disease-related variables								
Disease duration (years)		NA	90	5.2 (4.1)	66	7.1 (5.2)	NA	0.012*
Age at disease onset (years)		NA	90	56.8 (7.9)	66	61.5 (8.7)	NA	<0.001***
H&Y stage, median [IQR]		NA	90	2 [1.5- 2]	66	2 [2–2.5]	NA	0.049*
UPDRS I		NA	89	2.0 (1.8)	64	2.0 (1.6)	NA	0.843
UPDRS II		NA	89	9.4 (5.2)	65	10.1 (6.4)	NA	0.880
UPDRS III		NA	89	23.3 (10.7)	65	24.7 (12.0)	NA	0.452
UPDRS IV		NA	89	3.3 (3.2)	65	3.1 (3.5)	NA	0.344
LEDD (mg)		NA	90	592.2 (373.2)	66	627.7 (327.0)	NA	0.327
Cognitive outcomes								
MoCA^c^	71	26.1 (2.4)	88	24.3 (4.1)	66	22 (4.6)	0.001***	0.001***
Benton Line Orientation Judgment	65	23.1 (4.1)	72	21.4 (5.7)	50	17.6 (5.5)	0.049	0.002**
Salthouse Perceptual Comparison	67	24.7 (8.4)	73	23.5 (8.2)	51	16.6 (7.3)	0.402	0.001***
Symbol Digit Modality Test	67	45.6 (10.8)	72	35.5 (12.6)	50	26.2 (12.2)	<0.001***	<0.001***
Trail Making Test, part-A^d^	67	38.5 (11.7)	73	47.4 (22.5)	51	63.4 (29.2)	0.004**	0.001***
Trail Making Test, part-B^d^	57	89.0 (32.0)	67	124.5 (72.8)	49	184.1 (89.4)	<0.001***	<0.001***
Modified Wisconsin Card Sorting	56	5.2 (1.7)	59	4.4 (2.0)	34	3.3 (2.2)	0.022*	0.026
Primary visual outcomes								
High Contrast Visual Acuity^e^	35	63 (4.4)	44	60.8 (5.3)	28	59.1 (5.4)	0.048	0.103
High Contrast Visual Acuity^f^	36	44.3 (3.8)	42	43.6 (4.8)	35	41.4 (4.4)	0.470	0.114
Low Contrast Visual Acuity^e^	34	38.9 (6.7)	44	30.2 (10.3)	28	25.8 (11.7)	<0.001***	0.054
Low Contrast Visual Acuity^f^	36	29.4 (6.5)	42	24.7 (9.9)	35	20.4 (9.7)	0.016*	0.145
Contrast Sensitivity, photopic^g^	34	2.05 (0.14)	44	1.95 (0.15)	28	1.84 (0.1)	0.006**	<0.001***
Contrast Sensitivity, mesopic^g^	34	1.77 (0.1)	44	1.7 (0.1)	28	1.59 (0.17)	0.004**	0.003**
Contrast Sensitivity, contrast %^d, f^	34	1.09 (0.29)	42	1.6 (1.29)	35	2.11 (1.11)	0.020*	0.149
Contrast Sensitivity, no. of letters^f^	34	73.97 (1.88)	42	65.1 (19.7)	35	66.0 (12.8)	0.006***	0.808

*H&Y* Hoenh & Yahr scale, *LEDD* levodopa-equivalent daily dose, *MoCA* Montreal Cognitive Assessment, *NA* not applicable, *pfGCIPL* parafoveal ganglion cell-inner plexiform layer complex.

^a^The unadjusted p-values for baseline differences between controls and high pfGCIPL PD subgroup.

^b^The unadjusted p-values for baseline differences between high pfGCIPL and low pfGCIPL PD subgroup. No multiple comparison correction was applied for disease-related variables.

The significance level was set as *p* = 0.025 to control for multiple comparisons. Asterisks highlight the significant differences after Bonferroni correction as *p < 0.05, **p < 0.01, ***p < 0.001.

^c^Global cognition was assessed with MoCA, a validated instrument whose scoring system ranges from 0 to 30. Scores below 26 are indicative of mild cognitive impairment.

^d^Higher scores indicate worse performance (i.e., more seconds to complete the task or more contrast needed to identify letters).

Due to a protocol change, primary visual function was measured differently:

^e^Retro-illuminated cabinet at 4 meter using 100% contrast ETDRS and 2.5% contrast Sloan charts.

^f^Precision Vision Visual Acuity Test (PVVAT) digital software at 4m.

^g^Pelli-Robson Test at 1 m.

### Association between pfGCIPL thinning rate, progression of cognitive decline and disease stage

Longitudinally, the changes in pfGCIPL thickness did not temporally correlate with changes in clinical scores (Supplementary Fig. 3). However, PD patients with low baseline pfGCIPL thickness and slower pfGCIPL thinning rate had a steeper decline in MoCA score. Concretely, they showed a significant worsening of global cognition over time (*β* [SE] = −0.45 [0.19] points/year, *p* = 0.021) that was three times higher compared to PD patients with high pfGCIPL (*β* [SE] = −0.14 [0.11] points/year, *p* = 0.200). Nonetheless, individual cognitive and visual tests revealed that the differences between pfGCIPL thinning patterns were non-significant (Supplementary Table 6).

**Fig. 2:**
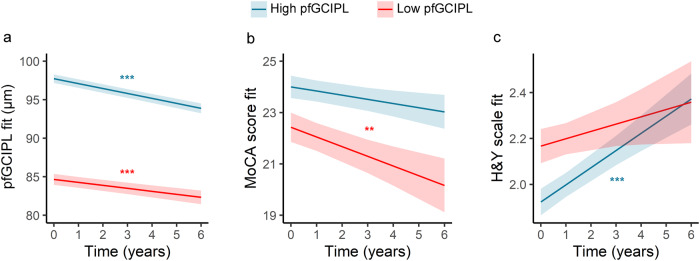
Progression of pfGCIPL thinning, global cognition and H&Y stage in PD patients. PD patients were divided into two subgroups based on their baseline pfGCIPL thickness (cut-off 89.8 µm), and the rates of pfGCIPL thinning (a), global cognition deterioration (b) and H&Y stage progression (c) were assessed. Parameter estimates from linear mixed-effect models were converted to and plotted as condition means and standard error. Asterisks indicate the significance of time from LMM within each PD subgroup (** p < 0.01, *** p < 0.001). *Abbreviations:* H&Y, Hoenh & Yahr scale; PD, Parkinson’s disease; MoCA, Montreal Cognitive Assessment; pfGCIPL, parafoveal ganglion cell-inner plexiform layer.

On the other hand, the progression of H&Y stage and motor impairment (UPDRS III) was significant for PD patients with fast pfGCIPL thinning rate (*β* [SE] = 0.08 [0.02] points/year, *p* < 0.001 and 1.24 [0.36] points/year, *p* < 0.001, respectively) but not for slow progressing ones (*β* [SE] = 0.03 [0.04] points/year, *p* = 0.356 and 1.27 [0.72] points/year, *p* = 0.084, respectively; Fig. 2), despite the non-significant interaction terms. A similar trend was observed for non-motor symptoms and the motor-related activities of daily living parts of the UPDRS (Supplementary Table 6).

### pRNFL thinning and its association with clinical progression

To deepen our understanding of the dynamics of retinal changes in PD, we examined the thickness of pRNFL in the test dataset. At baseline, both average pRNFL thickness and its sector-specific thicknesses showed no significant differences between PD patients and controls (Table 4). However, paralleling macular findings, pRNFL thickness demonstrated a more marked decline over time in PD patients compared to controls (*β* [SE] _time × group_ = −0.27 [0.40], *p* = 0.040). This was primarily attributed to an increased thinning in the temporal sector of PD patients (*β* [SE] _time × group_ = −0.67 [0.26], *p* = 0.009). Indeed, longitudinal changes in pfGCIPL thickness significantly correlated with changes in pRNFL thickness (*β* [SE] = 0.35 [0.08], *p* < 0.0001).

**Table 4:** Group differences in pRNFL baseline thickness and annualised thinning rate.

	*β*_group_ (SE)	*p* value	*β*_interaction_ (SE)	*p* value
pRNFL, mean	0.27 (1.40)	0.846	−0.26 (0.13)	0.040
pRNFL, superior	−0.11 (1.58)	0.946	−0.35 (0.18)	0.057
pRNFL, inferior	1.43 (2.14)	0.506	−0.01 (0.27)	0.968
pRNFL, nasal	−0.31 (2.11)	0.882	0.05 (0.14)	0.744
pRNFL, temporal	0.04 (2.32)	0.985	−0.67 (0.26)	0.009

*ß*_group_ represents the estimated baseline difference coefficient between PD (*n* = 155) and controls (*n* = 67) derived from age- and sex-adjusted LMMs. *ß*_interaction_ indicates the difference in the annualised estimated thinning rate in PD compared to controls.

*pRNFL* peripapillary nerve fiber layer, *SE* standard error.

Despite these trends, the covariances of pRNFL thickness (both average and by sectors) and clinical scores - including MoCA, UPDRS III, or H&Y - were not significantly correlated (Supplementary Fig. 4). Nonetheless, the association between changes in temporal pRNFL thickness and changes MoCA score approached significance when controlling for the effect of age, sex and within subject correlation (LMM, *β* [SE]_pRNFL, temporal_ = 0.11 [0.05], *p* = 0.052), suggesting a potential concurrent temporal progression.

Finally, subgroup analyses within the PD cohort revealed a significantly thinner pRNFL in all sectors in the low pfGCIPL subgroup compared to high pfGCIPL subgroup. However, there were no significant differences in the rate of pRNFL thinning over time between PD subgroups (Supplementary Table 7).

## Discussion

This longitudinal cohort study revealed that the rate of retinal thinning was significantly higher in PD patients compared to controls, particularly in pfGCIPL and the temporal sector of the pRNFL. Our findings indicate that the rate of retinal neurodegeneration varies among individuals with PD. Specifically, PD patients with greater baseline pfGCIPL atrophy exhibited slower rates of pfGCIPL thinning over time. These individuals had longer disease duration and greater disease severity, as assessed by cognitive (MoCA) and motor (H&Y scale) evaluations. Interestingly, in this group of severe PD patients with initial retinal atrophy and slower pfGCIPL thinning, cognitive decline progressed significantly faster than in other PD patients. This finding highlights a decoupled progression between macular changes and cognitive decline, suggesting that macular neurodegeneration may precede cognitive deterioration. Therefore, we interpret that once a certain threshold of retinal macular atrophy is reached, there is a potential deceleration in its pattern of degeneration. This deceleration reflects a higher disease severity, which is accompanied by an acceleration of cognitive impairment progression. Conversely, thinning in the temporal sector of pRNFL exhibited a close association with alterations in MoCA score, suggesting a simultaneous progression that could potentially serve as a valuable indicator for monitoring cognitive decline.

Prior cross-sectional studies investigating the relationship between retinal OCT and clinical outcomes in PD have demonstrated that inner retinal layer thickness is associated with disease duration and motor disability^16,17^. Also, thinning of GCIPL has been linked to dopaminergic loss in the *substantia nigra* in de novo PD patients^18^. This observation could partially explain the slower rate of GCIPL atrophy in PD patients with more pronounced cognitive impairment, as cognitive processes involve brain areas beyond the basal ganglia. Despite this, the tight relationship between retinal thickness and cognition is increasingly acknowledged^2,4,19^, even in early stages of the disease^3^. Consequently, the predictive nature of retinal imaging for cognitive outcomes is under extensive investigation. However, the ongoing exploration of the predictive potential of retinal imaging for cognitive outcomes reveals several unaddressed fundamental issues in the research field. For instance, previous longitudinal OCT studies in PD failed to assess specifically the rate of GCIPL and pRNFL thinning^6,20–22^. In this work, we expand upon our previous findings^2^ by demonstrating significantly higher rate of not only pfGCIPL but also pRNFL thinning in PD. Overall, the validation of our primary outcome in an external OCT dataset^23^ provides further evidence of accelerated retinal neurodegeneration in PD compared to controls. It is important to acknowledge that the OCT images from the validation cohort were obtained from a clinical setting, which implies certain limitations in terms of image quality control criteria^24^. Not only could the involvement of multiple operators using different OCT devices have influenced the final morphometric results^25^, but there is also a possibility that individuals with ophthalmic conditions were included in the control group of the validation cohort, potentially confounding our results. Additionally, there were some discrepancies between our dataset and the AlzEye dataset, such as shorter follow-up intervals and total follow-up period, as well as older mean age of patients and controls compared to our cohort and variation in the proportion of racial backgrounds. It is plausible that the variability within subjects and the inherent noise within the dataset may have contributed to some of the differences observed in the final results and to the lack of statistically significant interaction term between PD pfGCIPL subgroups. Despite these limitations, the estimated annual rates in different groups were successfully replicated in the AlzEye dataset, enhancing the robustness of our observations. Overall, our results indicate the presence of accelerated retinal neurodegeneration in PD patients, but the rate of pfGCIPL thinning appears to exhibit heterogeneity among patients. Unfortunately, due to the lack of enough OCT acquisitions of pRNFL in AlzEye, the results obtained for this area could not be replicated in the validation dataset.

In a second step, we aimed to explore the clinical correlates of pfGCIPL thinning rate patterns. Recently, Hannaway et al. ^6^ performed a 3-year longitudinal study and observed that pfGCIPL thinning did not show a linear relationship with cognitive worsening, establishing that the temporal dynamics were not associated. A notable finding in our study is the association between initial pfGCIPL atrophy and the subsequent slower rate of pfGCIPL thinning but more rapid decline in global cognition in PD. This intriguing relationship prompts a biological explanation for the seemingly contradictory phenomenon of slower retinal neuronal loss being linked to faster cognitive deterioration in PD. It is plausible that patients with more pronounced baseline pfGCIPL atrophy have already experienced a substantial depletion of their neuronal pool that may contribute to an accelerated decline in cognitive abilities, outpacing those with high baseline pfGCIPL thickness and better-preserved cognitive functions. Indeed, cognitive and functional disability of PD patients with slow rates of pfGCIPL thinning were remarkably worse compared to PD patients with accelerated retinal tissue loss in the macula, although all patients exhibited altered cognitive and visual abilities compared to controls. Furthermore, the significant rise in H&Y staging among fast pfGCIPL progressors warrants attention. Despite both groups having a median score of 2, the distribution of H&Y staging varied among pfGCIPL subgroups, with fast pfGCIPL progressors displaying milder impairment. We posit that, over the follow-up period, in the group with initially milder H&Y staging, the likelihood of progressing to the next stage was higher. This speculation is grounded in the notion that the transition from unilateral to bilateral impairment occurs more rapidly compared to advancing from bilateral to bilateral with postural instability stage^26^ .

Thus, our findings suggest that retinal thinning in the pfGCIPL area is likely a phenomenon that precedes advanced stages of PD, and therefore, it is not synchronous with cognitive or motor changes. This hypothesis is supported by cross-sectional observations reporting GCIPL thinning in de novo PD patients^3^ and in prodromal phases of Lewy body disease^27^. A similar phenomenon occurs in the *substantia nigra* of PD patients where an initial depletion of dopaminergic neurons takes place before motor symptoms become apparent^28^. Additionally, we observed an accelerated rate of thinning of temporal pRNFL in PD patients compared to controls. The temporal pRNFL sector primarily corresponds to the papillomacular bundle, where the majority of axons of ganglion cells from the macular area converge at the optic nerve. It has been suggested that, while pfGCIPL atrophy begins in the early stages, the temporal sector of the pRNFL is subsequently altered in middle-stage PD patients^29^. Despite not reaching statistical significance, there is an intriguing indication that temporal pRNFL thinning might parallel cognitive deterioration over time. Overall, our findings suggest that the pfGCIPL atrophy may reflect the underlying brain degeneration that precedes significant cognitive decline, making it suitable as a non-invasive biomarker to forecast the rate at which cognition will deteriorate over time, whereas monitoring changes in temporal pRNFL could be a promising avenue for further investigation as a potential biomarker to track cognitive decline.

The major limitation of the current study is the relatively short follow-up time, a limited number of follow-up visits per participant, and an inconsistent follow-up schedule across subjects. These factors restricted our ability to effectively track changes in pfGCIPL and pRFNL thinning within the same individual over a long period. Furthermore, our PD and control cohorts were not matched for age and sex. As both variables correlate with OCT measurements^30,31^ we accounted for these confounding variables in the statistical analyses. Moreover, the absence of pRNFL measurements and clinical data in the AlzEye dataset hindered our ability to validate all of our analyses in an external database. Therefore, it remains uncertain whether there is a true alteration in the rate of retinal thinning over time within the same individual, which could potentially indicate a change in the progression of the disease. It should be noted that individuals with PD were categorized into two groups as a solution to address the limitation of insufficient follow-up data. Given the potential complexity of retinal dynamics, our simplified approach might have resulted in the absence of significant interaction terms in certain models, as it might disregard a potential continuum. Moreover, cognitive decline in PD patients might also be influenced by Alzheimer’s co-pathology. The lack of patient stratification according to cerebrospinal fluid or plasma biomarkers of Alzheimer’s disease is a significant limitation of the current study. Finally, the annualised retinal changes are below the resolution limit of OCTs and advancements in OCT technology may be required before our findings are escalated into clinics. Nonetheless, we believe that our findings lay the groundwork for future research investigating the dynamics of retinal changes and their relationship with cognition and disease progression in PD.

A strength of our study lies in its multicenter design with a robust sample size, which includes an independent validation cohort. Our findings shed light on the intricate nature of the relationship between changes in OCT metrics and cognitive deterioration. The consistency of our estimations across diverse geographic locations and various OCT devices enhances the generalisability of our results, and underscore the potential value of longitudinal macular OCT scans for assisting clinical decision-making for PD-related cognitive decline. However, it is imperative to conduct studies with more comprehensive longitudinal data and extended follow-up periods to establish the robustness and reliability of our findings.

## Methods

### Study design and participants

The study was based on the analysis of 2 longitudinal databases. The test dataset included participants prospectively enrolled between February 2015 and December 2021 in two movement disorder units in Spain, 156 patients with idiopathic PD (130 in Cruces University Hospital and 26 in Araba University Hospital) and 72 controls (all in Cruces University Hospital). 49 PD patients and 27 controls had only baseline assessment. Participants with follow-up visits underwent two to three assessments at varying time intervals (ranging from 1 to 5 years). Some participants had visits at baseline, 1-year, and 2-years, while others had visits at baseline, 3-years, and 5-years, or baseline and 2-years as indicated in Supplementary Fig. 1. The total number of observations was 304 for PD and 141 for controls. Patients with PD met the criteria established by the Parkinson’s UK Brain Bank. We excluded patients from the study if they tested positive for PD-causing genetic mutations during routine clinical care. This testing is conducted for individuals with more than one first or second-degree relative affected by the disease or those under 50 years of age. Given our geographic context, the standard panel includes screening for mutations in LRRK2, PARK2, and SNCA. Controls were not included if they had more than one first-degree relative with a PD diagnosis or manifestations suggestive of PD. A screening protocol was performed in all participants to exclude subjects with potential confounding factors influencing clinical outcomes or retinal OCT measures, as previously described^2^. This includes individuals with eye diseases such as corneal opacities, severe cataracts, and retinal alterations like glaucoma, macular degeneration, or history of any retinal pathology. Participants with cataracts or corneal alterations that did not compromise the quality of OCT scans, as per the OSCAR-IB criteria^24^, were considered eligible. Additionally, those with a history of inflammatory eye diseases, consumption of drugs known to induce retinal pathology or affect media transparency, chronic systemic inflammatory diseases, a diagnosis of diabetes mellitus, uncontrolled hypertension, a history of brain trauma, or any neurological disorder other than PD were ineligible. Participants with a history of severe smoking or heavy alcohol use were also excluded.

Age, sex, and education years were recorded in all participants. In PD patients, we recorded age at disease onset, disease duration at baseline, Hoehn & Yahr (H&Y) score, Unified Parkinson’s disease Rating Scale (UPDRS), and L-dopa equivalent daily dose (LEDD). Patients were not excluded based on age or disease duration. The study was approved by Comité Ético de Investigación Clínica – Euskadi (study code: PI2020025), and all participants gave written informed consent.

The validation dataset consisted of an external record-level longitudinal OCT dataset from Moorfields Eye Hospital NHS Foundation Trust collected for the AlzEye study^23^. In the validation cohort, OCTs from 873 controls and 167 PD patients were selected. Among them, 361 controls (41.4%) and 73 PD patients (43.7%) had at least two visits with a mean [SD] follow-up period of 1.4 [1.6] and 1.3 [1.4] years, respectively. The majority (>70%) belonged to the white racial group (Supplementary Table 2). The inclusion and exclusion criteria for the validation dataset are described in Supplementary Methods (Supplementary Fig. 5). This retinal OCT database contained exclusively demographic information (age and sex) and diagnostic labels based on ICD-10 coding. The AlzEye study dataset was used to validate the results referring to the macular OCT changes in PD and controls, and not to analyze the associations between OCT and clinical variables. The AlzEye study has received institutional and ethical review board approval, including an exemption of participant consent (REC reference. 18/LO/1163).

### Cognitive and visual function assessment

Clinical assessments included cognitive disability scales and visual function assessment as reported before^1,2,32^. Global cognition was tested with Montreal Cognitive Assessment (MoCA). Visuospatial ability was measured with Benton Line Orientation Judgment (BLOJ). Visual attention and processing speed was assessed with Salthouse Perceptual Comparison Test (SPCT), Symbol Digit Modality Test (SDMT) and Trail Making Test, part-A (TMT-A). Executive functions were tested with Trail Making Test, part-B (TMT-B) and Modified Wisconsin Card Sorting Test.

A comprehensive visual screening protocol was performed for participant inclusion and primary visual function was evaluated with validated tests. Primary visual function was measured binocularly with best-corrected refraction. High-contrast visual acuity and low-contrast visual acuity were registered as the total number of letters correctly identified at 4 meters. Due to a change in protocol, visual acuity was measured with 100% contrast Early Treatment Diabetic Retinopathy Study (ETDRS) charts and Sloan 2.5% contrast charts (Precision Vision, La Salle, IL) mounted in a retro-illuminated cabinet in some participants, whereas in others it was measured with Precision Vision Visual Acuity Test (PVVAT) digital software. Similarly, contrast sensitivity was measured with a Pelli-Robson chart at 1 m under photopic (280 lux) and mesopic (1.5 lux) conditions, and the lowest contrast at which 2 letters in a triplet were correctly identified was recorded. In others, contrast sensitivity was measured with PVVAT digital software, and the lowest contrast that could be identified as well as the number of correctly identified letters were recorded. Note that higher contrast in contrast sensitivity measurement reflects worse function.

### Optical coherence tomography acquisition

Image acquisition and preprocessing was different for each dataset. Cruces University Hospital and Araba University Hospital images were acquired with a Spectralis SD-OCT device (Heidelberg Engineering, Heidelberg, Germany) without pupil dilation by four graders using the same acquisition protocol, as previously described^1,2^. A macular raster scan protocol was used with 25 horizontal B-scans and 512 A-scans per B-scan covering a 6 × 6 mm^2^ region. For each final B-scan, 49 slices were averaged to improve image quality. Peripapillary nerve fiber layer (pRNFL) OCTs were acquired using a 3.5 mm circular scan manually centered in the optic nerve, with 100 images per B-scan obtained through the Automatic Real Time-function mode (ART = 100). Subsequent images of the same eye were acquired using the follow-up Spectralis function, which ensures that the imaged region is the same across visits. The images were automatically segmented by HEYEX algorithm (versions 1.10.4.0 and 1.12.1.0), and were manually inspected to detect image quality problems and correct minor segmentation errors. Images with considerable segmentation errors were discarded. The foveal center was automatically located as the minima of the smoothed Total Retinal Thickness map in macular raster scans. All included OCT scans fulfilled OSCAR-IB criteria^24^.

For the validation dataset (AlzEye project), OCTs were acquired by several graders and with different Topcon OCTs (Topcon Corporation, Tokyo, Japan) as indicated in Supplementary Table 8. A macular cube protocol was used covering a 6 × 6 mm^2^ region with 128 horizontal B-scans and 512 A-scans per B-scan. All the images were segmented by Topcon Advanced Boundary Segmentation algorithm^33^. This algorithm is based on dual-scale gradient graph search and provides both layer segmentation and foveal center coordinates. AlzEye OCT data curation is detailed in Supplementary Fig. 5. Included OCT images were manually inspected by two experienced operators.

In test dataset, pRNFL OCTs were acquired using a 3.5 mm circular scan manually centered in the optic nerve, with 100 images per B-scan obtained through the Automatic Real Time-function mode (ART = 100). The average pRNFL thickness and the thickness in each quadrant – superior, inferior, nasal, and temporal - were calculated. The AlzEye dataset did not include OCT acquisitions of the peripapillary region for most subjects. Consequently, the results obtained for this area could not be replicated in the validation dataset.

### Optical coherence tomography image processing

After preprocessing, an equivalent feature extraction pipeline was used for both datasets to evaluate the retinal layer thicknesses. This step was carried out separately in both MATLAB R2021b (Cruces University Hospital and Araba University Hospital) and Python 3.7.3 (AlzEye), and was based on the RETIMAT toolbox^34,35^. First, the located foveal center was set as the origin of coordinates. Then, point thickness values were interpolated into a 200 × 200 point regular grid covering the 6 ×6 mm^2^ region. Left eyes were flipped to match right eyes.

For the initial topographical analysis of retinal changes in the macula, point by point thicknesses were averaged across 5 concentric rings 0.5 mm apart, centered on the fovea. This sectorization of the macular area allowed us to explore subtle trends that could have been obscured by the broader ring sizes, i.e., the conventional 1-mm, 1- to 3-mm, and 3- to 6-mm rings from the ETDRS grid. In addition, we further divided the concentric rings into quadrants to exhaustively examine the data. However, our results revealed that: (1) The atrophy rate in PD versus control groups was most noticeable in the GCIPL; (2) Within the GCIPL, the parafoveal area exhibited a more pronounced rate of thinning; (3) Among the parafoveal concentric rings in the GCIPL, the 1- to 2-mm and 2- to 3-mm thicknesses showed the strongest correlation with clinical outcomes (see Supplementary Fig. 2 for an illustration); (4) Dividing the parafoveal rings into quadrants did not yield sufficient evidence of a distinct trend (in terms of atrophy rate or correlation with clinical outcomes) to justify splitting the macular parameters and analyzing them separately. After conducting this comprehensive analysis, we concluded that there was no substantial benefit to an exhaustive examination over using a more clinically practical approach, which also facilitates translatability, and minimizes the issue of multiple comparisons. As a result, the the 1- to 3-mm GCIPL thickness was computed and we refer to this parameter as the parafoveal GCIPL (pfGCIPL).

Based on the baseline pfGCIPL thickness, each group was divided into two subgroups. The selected cut-off value for this classification derived from the thickness distribution of a reference control sample with a comparable age range (Supplementary Methods, Supplementary Fig. 6), and the upper limit of the lowest quartile of pfGCIPL thickness was used as the cut-off value^2^. In test dataset, a cut-off of 89.8 µm was used (reference population: n = 415 controls) and 78.8 µm in validation dataset (reference population: n = 873 controls). The results are reported adhering to the APOSTEL 2.0 recommendations^36^.

Obtained thickness values were averaged between left and right eyes to obtain a single value per subject and visit. When only one eye was included, the thickness value of that eye was used.

### Statistical analysis

We described baseline features of the study population using absolute and relative frequencies for categorical variables, and mean and standard deviation for quantitative variables, unless otherwise stated. Group characteristics were compared using Chi-square or Fisher’s exact test for categorical variables and T-test or Wilcoxon test for quantitative variables, as appropriate. The Bonferroni p-value correction was applied when deemed appropriate to control for Type I error rate in multiple comparisons.

To estimate the annual rates of change, we fitted linear mixed models (LMM) using *lme4* and *lmerTest* packages^37,38^. We included a random intercept for subjects. First, we adjusted LMMs for PD and controls, separately, using fixed effects of years since baseline (time to follow-up), age at baseline, and sex. When the outcome variable was a cognitive measure, models were further adjusted for years of education. In a second step, models were fitted using as the main fixed effects time to follow-up, group, their interaction term (time *x* group). In view of the differences in age at baseline and sex between groups, LMMs were adjusted for these confounding factors. Years of education was also included in models where the outcome variable was cognitive. The normal distribution and homoscedasticity of residuals were examined to ensure that model assumptions were not violated. Data was assumed to be missing at random.

Statistical analyses were performed using RStudio (version 2022.07.0) and MATLAB R2021b from February 2023 to June 2023. The significance level was set at *α* = 0.05.

### Reporting summary

Further information on research design is available in the Nature Research Reporting Summary linked to this article.

### Supplementary information

Supplementary Information

Reporting Summary

## Data Availability

The datasets generated and/or analyzed during the current study are not publicly available due to ethical or legal restrictions but are available from the corresponding author on reasonable request.

## Code availability

The underlying code for this study is not publicly available but may be made available to qualified researchers on reasonable request from the corresponding author.
