# Supplementary material for: Association of retinal neurodegeneration with the progression of cognitive decline in Parkinson’s disease
Source: NPJ Parkinsons Dis. 2024 Jan 23;10:26. doi: 10.1038/s41531-024-00637-x (PMC10805713; doi:10.1038/s41531-024-00637-x)
Supplement: Supplementary file 1 — Supplementary Information [file 41531_2024_637_MOESM1_ESM.pdf]

Supplementary Information

Association of retinal neurodegeneration with the progression of cognitive decline in Parkinson's disease

Murueta-Goyena et al.

CONTENTS:

Supplementary Figures

Supplementary Figure 1. Study flow chart

Supplementary Figure 2. Baseline pfGCIPL correlations

Supplementary Figure 3. Temporal correlation of covariances between pfGCIPL and clinical outcomes

Supplementary Figure 4. Temporal correlation of covariances between temporal pRNFL and clinical outcomes

Supplementary Tables

Supplementary Table 1. Annualized rates of cognitive and visual deterioration in patients with PD

Supplementary Table 2. Description of the follow-up time and intervals from the validation dataset (AlzEye study)

Supplementary Table 3. Estimated annualized pfGCIPL thinning rate in PD further adjusting for disease duration

Supplementary Table 4. Adjusted baseline and rate differences in control participants from test dataset depending on the baseline pfGCIPL thickness

Supplementary Table 5. Characteristics of control participants and PD patients of validation dataset (AlzEye study) based on their initial pfGCIPL thickness

Supplementary Table 6. Adjusted baseline differences and progression rates between high and low pfGCIPL subgroups in PD

Supplementary Table 7. Baseline pRNFL thickness and annualized pRFNL thinning rate in PD patients depending on pfGCIPL subgroup

Supplementary Table 8. Topcon OCT devices used in AlzEye study and the corresponding number of images used in the current dataset

Supplementary Methods

Supplementary Figure 5. Validation cohort flow chart

Supplementary Figure 6. Box plots and flat violin plots of age (A) and pfGCIPL (B) distribution in the reference control sample

Supplementary Figures

Participants and Study Design from Test Dataset

In this longitudinal prospective study, participants were followed up at various time intervals ranging from 1 year to 5 years. Participants who completed follow-up visits had a total of 2 to 3 visits. Some participants had visits at baseline, 1 year, and 2 years, while others had visits at baseline, 3 years, and 5 years, or only at baseline and 2 years for follow-up.

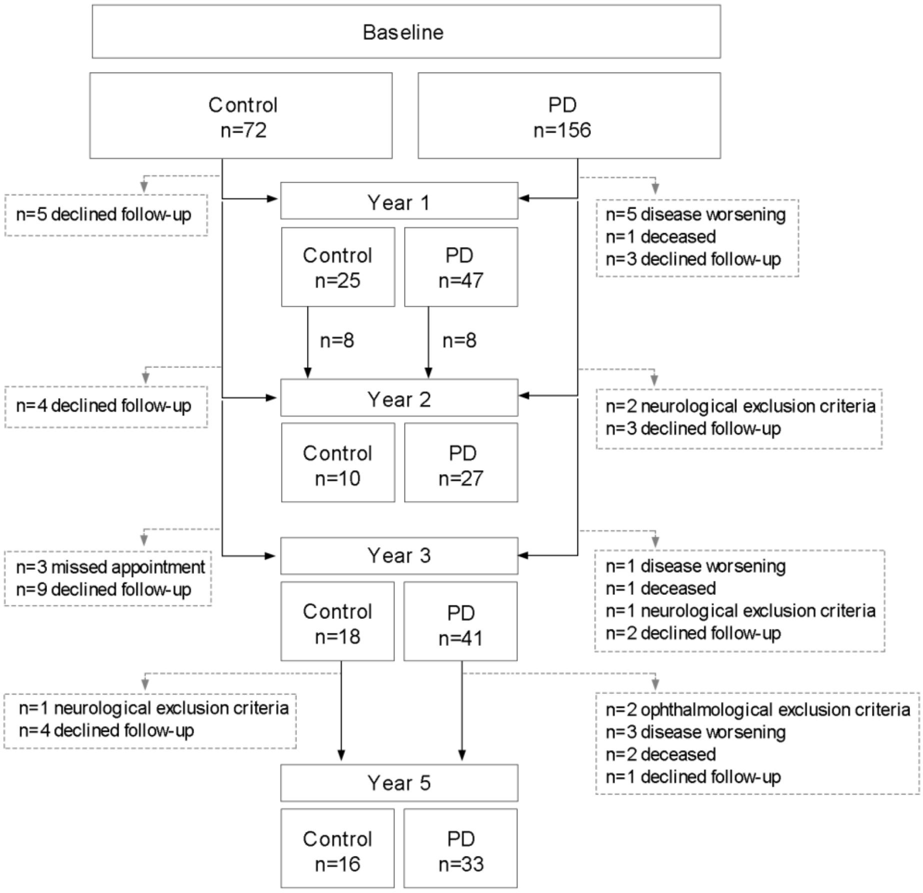

Supplementary Figure 1. Study flow chart. Description of the follow-up design of study participants. Lost to follow-up with reasons in each stage is represented with dashed arrows and boxed. *Abbreviations:* PD, Parkinson's disease.

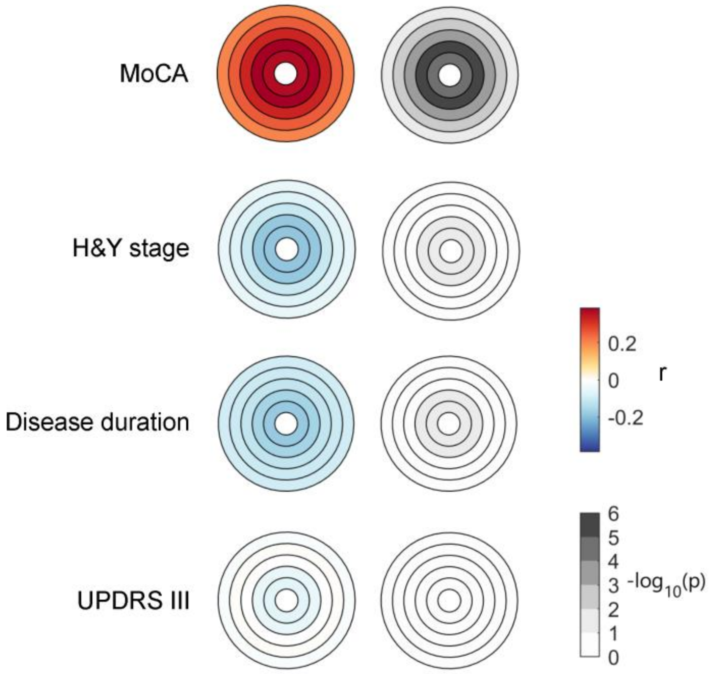

Supplementary Figure 2. Baseline pfGCIPL correlations in patients with Parkinson's disease

Pearson correlation coefficients (r) between pfGCIPL thickness and clinical outcomes are colour-coded and significant p-values are shown in grey scale. For calculating correlation coefficients, the thickness values of both eyes were averaged. *Abbreviations*: H&Y, Hoehn & Yahr scale; MoCA, Montreal Cognitive Assessment; pfGCIPL, parafoveal ganglion cell-inner plexiform layer; UPDRS, Unified Parkinson's disease Rating Scale.

Preliminary Analyses to Explore the Temporal Relationship between the Retina and Cognition

From all macular parameters, the pfGCIPL area was selected over the other sectors based on several key factors. Firstly, this region exhibited some of the largest absolute annualized changes. Secondly, it showed the highest correlation coefficients with clinical outcomes (sFig.2). Lastly, in the validation cohort the pfGCIPL thinning rate was the only GCIPL area with significantly higher atrophy rates in PD compared to controls. The average thickness of the peripapillary nerve fiber layer (pRNFL) was also used for exploring temporal association between the retina and clinical outcomes.

To assess the temporal association between changes in retinal measurements (i.e., pfGCIPL and pRNFL) and the progression in clinical outcomes, first, the correlation between the covariances was visually inspected. For that, we plotted all possible differences between consecutive visits for each patient. For example, if a patient had visits at year 0, year 1, and year 2, we calculated the differences between year 0 and year 1, year 1 and year 2, and year 0 and year 2. We then plotted a regression line to these differences. The temporally correlated covariance approached zero for pfGCIPL, as shown in sFig.5.

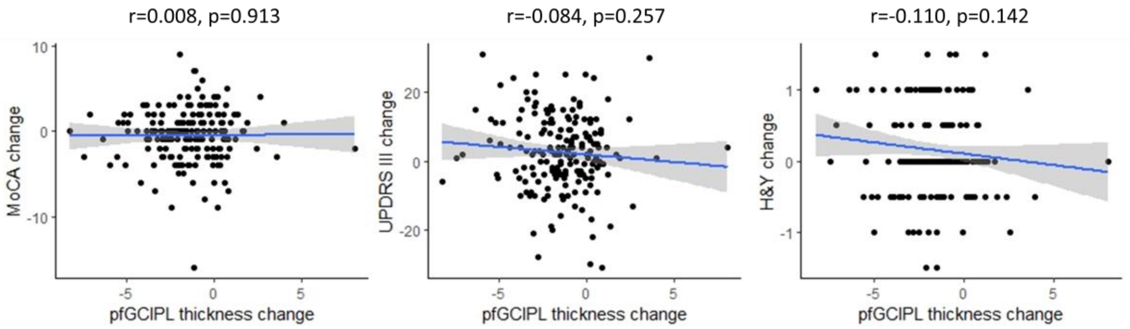

Supplementary Figure 3. Temporal correlation of covariances between pfGCIPL and clinical outcomes.

Furthermore, we conducted an additional exploratory analysis in our cohort with subjects who had OCT data available for three time points. These analyses revealed the model with a correlated random slope and intercept had a significantly better fit (Akaike Information Criterion, [AIC] = 635.1) than the model with uncorrelated random slope and intercept (AIC = 814.4), suggesting that baseline pfGCIPL thickness was significantly associated with the rate of pfGCIPL atrophy. Therefore, as an approach to explore the non-constant rate of pfGCIPL

atrophy within PD subjects, we divided participants based on their initial pfGCIPL measurements. To determine the cut-off value for this division, we used a reference control group consisting of 415 subjects (see Supplementary Methods). These subjects were recruited in the Neurology and Ophthalmology Departments of Cruces University Hospital between February 2015 and February 2023, and included participants from 40 to 80 years old (61.2% females).The upper limit of the lower quartile of the control group's pfGCIPL thickness (i.e., 89.8 µm) was selected as the cut-off value. We verified that the estimated rate of pfGCIPL atrophy (adjusted for age at baseline and sex) was significantly different between patients with pfGCIPL measures above or below 89.8 µm.

For pRNFL, a nearly significant correlation of covariances was observed for the temporal sector with MoCA scores and H&Y stage (sFig.4), but not for the remaining quadrants of average pRNFL thickness. To control for multiple observations in subjects and the influence of age and sex, linear mixed-effect models were fitted (results within the squares of sFig.4). Absolute changes in clinical scores were used as the outcome, with absolute changes in pRNFL (average and by sectors) serving as the main fixed effect. Additionally, age at baseline and sex were included as remaining fixed effects, and a random intercept for subjects was incorporated.

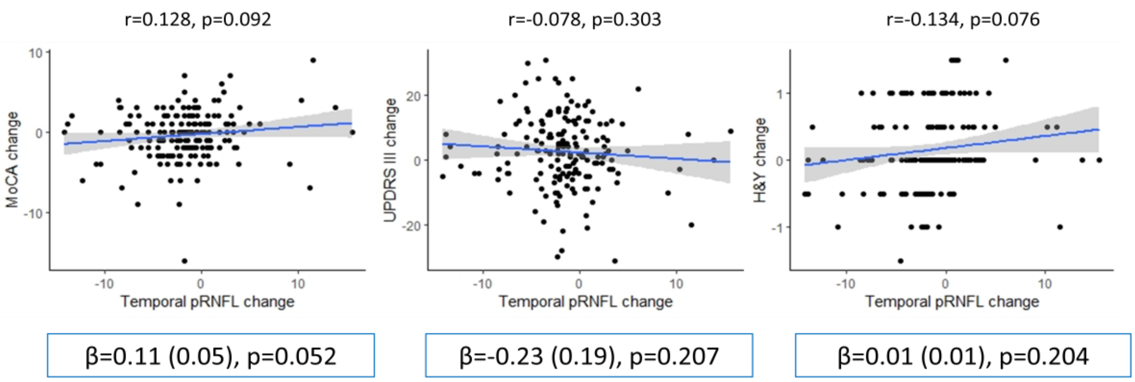

Supplementary Figure 4. Temporal correlation of covariances between temporal pRNFL and clinical outcomes. Results within the square are derived from linear mixed-effects models controlling for age at baseline, sex and within-subject correlation.

Supplementary Tables

Supplementary Table 1. Annualized rates of cognitive and visual deterioration in patients with PD

	Control				PD				Interaction term time *x* group		
	n	β_{time}	SE	p	n	β_{time}	SE	p	β_{int}	SE	p
Cognitive outcomes											
MoCA	72	0.04	0.10	0.675	154	-0.22	0.09	0.022	-0.19	0.16	0.232
Benton Line Orientation Judgment	68	-0.03	0.14	0.86	125	0.03	0.11	0.767	0.10	0.19	0.584
Salthouse Perceptual Comparison Test	70	-0.15	0.23	0.499	126	-0.9	0.15	<0.001 ***	-0.71	0.26	0.008 **
Symbol Digit Modality Test	70	-0.66	0.31	0.037 *	125	-1.05	0.23	<0.001 ***	-0.33	0.40	0.411
Trail Making Test, part-A [a]	70	1.68	0.61	0.007 **	126	1.89	0.57	<0.001 ***	0.05	0.93	0.960
Trail Making Test, part-B [a]	61	8.84	2.28	<0.001 ***	124	8.24	1.88	<0.001 ***	-1.6	3.2	0.616
Modified Wisconsin Card Sorting Test	60	-0.06	0.07	0.366	112	-0.05	0.06	0.388	0.01	0.10	0.880
Primary visual function											
High Contrast Visual Acuity [b]	35	-0.70	0.24	0.006 **	72	-0.58	0.19	0.003 **	0.13	0.32	0.672
High Contrast Visual Acuity [c]	36	0.06	0.41	0.882	98	-1.11	0.33	0.001 ***	-1.17	0.54	0.033 *
Low Contrast Visual Acuity [b]	34	-1.65	0.39	<0.001 ***	72	-1.99	0.29	<0.001 ***	-0.31	0.50	0.532
Low Contrast Visual Acuity [c]	36	-0.24	0.63	0.703	98	-1.01	0.59	0.089	-0.86	0.90	0.341
Contrast Sensitivity, photopic [d]	34	-0.03	0.01	<0.001 ***	72	-0.02	0.004	<0.001 ***	-0.00	0.00	0.923
Contrast Sensitivity, mesopic [d]	34	-0.02	0.01	<0.001 ***	72	-0.01	0.004	<0.001 ***	-0.00	0.00	0.140
Contrast Sensitivity, contrast %) [a, c]	36	-0.01	0.04	0.722	98	0.1	0.07	0.148	0.12	0.09	0.213
Contrast Sensitivity, number of letters [c]	36	-0.06	0.21	0.789	98	2.95	1.33	0.028	2.9	1.9	0.133

Annualized rates of change were calculated using LMM using time to follow-up as the main fixed effect and adjusted for age at baseline and sex. LMMs were further adjusted for years of education when the dependent variable was a cognitive score. To calculate the rates of change within groups, separate LMMs were fitted for controls and iPD. Next, an interaction term for time to follow-up and group was used as the main fixed effect to assess between group differences in the rates of change.

[a] Higher scores indicate worse performance (i.e., more seconds to complete the task or more contrast needed to identify letters).
Due to a protocol change, primary visual function was measured differently:
[b] Retro-illuminated cabinet at 4 meter using 100% contrast ETDRS and 2.5% contrast Sloan charts.
[c] Precision Vision Visual Acuity Test (PVVAT) digital software at 4m
[d] Pelli-Robson Test at 1m
Statistical significance: *p<0.05, ** p<0.01, *** p<0.001
Abbreviations: PD, Parkinson's disease; MoCA, Montreal Cognitive Assessment.

Supplementary Table 2. Description of the follow-up time and intervals from the validation dataset (AlzEye study)

	Control	PD
n at baseline	873	167
n with at least 1 follow-up	361	73
Follow-up time (years)		
Mean (SD)	1.4 (1.6)	1.3 (1.4)
Min-max	0 – 8.8	0 – 6.8
No. of visits		
Mean (SD)	4.4 (5.6)	4.7 (6.0)
Min - max	2 – 54	2 – 35
Age (years old)		
Mean (SD)	75.7 (9.4)	76.7 (8.6)
Min - max	37.3 – 100.7	44.7 – 92.3
Male sex, no. (%)	529 (60.6)	93 (55.7)
Race		
White, no. (%)	622 (71.3)	125 (74.9)
Asian or Asian British, no. (%)	105 (12.0)	21 (12.5)
Black or Black British, no. (%)	44 (5.0)	4 (2.4)
Mixed, no. (%)	100 (11.5)	16 (9.6)
Other ethnic groups, no. (%)	2 (0.2)	1 (0.6)

Abbreviations: PD, Parkinson's disease

Supplementary Table 3. Estimated annualized pfGCIPL thinning rate in PD further adjusting for disease duration

	n	$ß_{time}$ (SE)	p	$ß_{interaction}$ (SE)	p
**Outcome: pfGCIPL thickness**					
PD, high pfGCIPL	90	-0.65 (0.07)	<0.001	0.25 (0.12)	0.042
PD, low pfGCIPL	66	-0.38 (0.10)	<0.001		
**Outcome: MoCA**					
PD, high pfGCIPL	90	-0.14 (0.11)	0.180	-0.22 (0.21)	0.306
PD, low pfGCIPL	66	-0.46 (0.19)	0.020		
**Outcome: H&Y**					
PD, high pfGCIPL	90	0.08 (0.02)	<0.001	-0.05 (0.04)	0.186
PD, low pfGCIPL	66	0.04 (0.04)	0.319		

$ß_{time}$ represents the estimated coefficient of the annualized pfGCIPL thinning rate (μm/year) derived from age, sex and disease duration adjusted LMMs. $ß_{interaction}$ indicates the increased thinning rate in PD or high pfGCIPL subgroup compared to controls or low pfGCIPL subgroup, respectively. *Abbreviations*: PD, Parkinson's disease; pfGCIPL, parafoveal ganglion cell-inner plexiform layers; SE, Standard Error.

Supplementary Table 4. Adjusted baseline and rate differences in control participants from test dataset depending on the baseline pfGCIPL thickness

	Baseline			Rate of progression	
	n	β_{group} (SE)	p	$\beta_{time\ x\ group}$ (SE)	p
OCT-derived measures					
pfGCIPL	72	-11.4 (1.2)	<0.001***	0.004 (0.14)	0.977
Cognitive outcomes					
MoCA	71	-0.63 (0.57)	0.274	0.17 (0.25)	0.501
Benton Line Orientation Judgment	65	-0.03 (0.97)	0.974	-0.37 (0.34)	0.273
Salthouse Perceptual Comparison Test	67	-1.3 (1.6)	0.423	0.09 (0.54)	0.864
Symbol Digit Modality Test	67	-2.7 (2.3)	0.144	0.89 (0.73)	0.231
Trail Making Test, part-A [a]	67	-5.9 (3.5)	0.100	2.4 (1.4)	0.088
Trail Making Test, part-B [a]	57	-8.0 (11.1)	0.479	11.2 (5.2)	0.036*
Modified Wisconsin Card Sorting Test	56	-0.75 (0.46)	0.107	0.00 (0.16)	0.997
Primary visual functions					
High Contrast Visual Acuity [b]	29	-1.68 (2.22)	0.455	-0.30 (0.66)	0.646
High Contrast Visual Acuity [c]	33	-0.07 (1.14)	0.954	0.08 (0.91)	0.927
Low Contrast Visual Acuity [b]	29	-2.69 (3.9)	0.491	0.31 (1.03)	0.765
Low Contrast Visual Acuity [c]	33	1.65 (2.0)	0.416	-0.41 (1.35)	0.765
Contrast Sensitivity, photopic [d]	29	-0.03 (0.06)	0.637	0.002 (0.014)	0.893
Contrast Sensitivity, mesopic [d]	29	0.03 (0.06)	0.467	-0.010 (0.012)	0.415
Contrast Sensitivity, contrast % [c]	33	-0.15 (0.11)	0.165	-0.011 (0.09)	0.901
Contrast Sensitivity, number of letters [c]	33	0.58 (0.58)	0.322	-0.31 (0.43)	0.487

Annualized estimates were calculated using LMM adjusted for age at baseline and sex. The model was further adjusted for years of education when the dependent variable was a cognitive score. Estimates of baseline differences were derived from the ß estimation for group factor and estimates for differences in the rate of progression from ß estimation for the interaction term between time to follow-up and group. iPD group with low baseline pfGCIPL thickness (<89.8 µm) was selected as the reference group.

[a] Higher scores on the TMT indicate seconds to complete the task (i.e., worse performance).
Due to a protocol change, primary visual function was measured differently:
[b] Retro-illuminated cabinet at 4 meter using 100% contrast ETDRS and 2.5% contrast Sloan charts.
[c] Precision Vision Visual Acuity Test (PVVAT) digital software at 4m
[d] Pelli-Robson Test at 1m
*Statistically significant: * p<0.05, *** p<0.001
Abbreviations: MoCA, Montreal Cognitive Assessment; pfGCIPL, parafoveal ganglion cell-inner plexiform layer; SE, Standard Error.

Supplementary Table 5. Characteristics of control participants and PD patients of validation dataset (AlzEye study) based on their initial pfGCIPL thickness

	Control		PD	
	High pfGCIPL	Low pfGCIPL	High pfGCIPL	Low pfGCIPL
n	655	218	115	52
No. follow-up	273	88	48	25
Age, mean (SD)	74.7 (9.6)	78.7 (8.4)	75.3 (8.8)	79.6 (7.2)
Male, no. (%)	395 (60.3)	134 (61.5)	61 (53.0)	32 (61.5)
Disease duration (years) *	NA	NA	2.5 (2.4)	3.1 (2.6)

The threshold for defining baseline high or low pfGCIPL thickness was calculated as the upper limit of the lowest quartile in the control group. The cut-off pfGCIPL value was 78.7 µm. “No. follow-up” shows the number of participants with at least one follow-up visit from the included subjects. * Disease duration from AlzEye dataset was calculated as the time interval between the baseline OCT visit and the time point were the first tag of “Parkinson’s disease” appears in the dataset. *Abbreviations:* NA, not applicable; PD, Parkinson’s disease; pfGCIPL, parafoveal ganglion cell-inner plexiform layer.

Supplementary Table 6. Adjusted baseline differences and progression rates between high and low pfGCIPL subgroups in PD

	Baseline differences		Differences in the rate of change					
			High pfGCIPL		Low pfGCIPL			
	β_{group} (SE)	p	β_{time} (SE)	p	β_{time} (SE)	p	β_{int}(SE)	p
Disease-related outcomes								
H&Y stage, median [IQR]	0.24 (0.10)	0.012 *	0.08 (0.02)	<0.001 ***	0.03 (0.04)	0.356	-0.04 (0.04)	0.261
UPDRS I	-0.06 (0.31)	0.843	0.17 (0.07)	0.018 *	0.19 (0.10)	0.070	0.05 (0.13)	0.701
UPDRS II	0.14 (0.97)	0.880	0.67 (0.16)	<0.001 ***	0.41 (0.28)	0.132	-0.15 (0.31)	0.621
UPDRS III	0.81 (1.83)	0.658	1.24 (0.36)	<0.001 ***	1.27 (0.72)	0.084	0.01 (0.72)	0.993
UPDRS IV	0.52 (0.54)	0.344	0.26 (0.12)	0.034 *	0.30 (0.14)	0.044 *	0.10 (0.21)	0.623
LEDD (mg)	39.3 (60.0)	0.514	26.6 (10.8)	0.015 *	38.5 (16.01)	0.020 *	16.1 (19.1)	0.411
Cognitive outcomes								
MoCA	-1.57 (0.74)	0.035 *	-0.14 (0.11)	0.201	-0.46 (0.19)	0.020 *	-0.22 (0.21)	0.307
Benton Line Orientation Judgment	-1.16 (0.93)	0.217	0.13 (0.14)	0.349	-0.24 (0.17)	0.158	0.01 (0.33)	0.230
Salthouse Perceptual Comparison Test	-3.24 (1.17)	0.006 **	-0.92 (0.17)	<0.001 ***	-0.88 (0.29)	0.004 **	0.48 (0.51)	0.976
Symbol Digit Modality Test	-4.43 (1.98)	0.027 *	-1.15 (0.27)	<0.001 ***	-0.84 (0.4)	0.042 *	3.9 (4.2)	0.340
Trail Making Test, part-A [a]	7.44 (4.22)	0.080	2.1 (0.64)	0.001 ***	1.45 (1.18)	0.223	-1.0 (1.3)	0.437
Trail Making Test, part-B [a]	36.83 (13.80)	0.009 **	7.11 (2.19)	0.002 **	12.44 (3.49)	<0.001 ***	-0.3 (0.2)	0.351
Modified Wisconsin Card Sorting Test	-0.33 (0.40)	0.408	0.01 (0.06)	0.896	-0.18 (0.13)	0.174	-0.06 (0.15)	0.118
Primary visual function								
High Contrast Visual Acuity [b]	-0.11 (1.24)	0.937	-0.51 (0.22)	0.024 *	-0.82 (0.36)	0.030 *	0.27 (0.41)	0.521
High Contrast Visual Acuity [c]	-0.41 (1.01)	0.689	-0.84 (0.36)	0.023 *	1.54 (0.59)	0.012 *	-0.83 (0.68)	0.224
Low Contrast Visual Acuity [b]	-1.60 (2.69)	0.553	-1.81 (0.33)	<0.001 ***	-2.58 (0.60)	<0.001 ***	-0.73 (0.64)	0.258
Low Contrast Visual Acuity [c]	0.11 (2.12)	0.960	0.01 (0.74)	0.983	-2.38 (0.88)	0.010 **	-2.27 (1.16)	0.055
Contrast Sensitivity, photopic [d]	-0.06 (0.03)	0.063	-0.03 (0.01)	<0.001 ***	-0.01 (0.00)	0.006 **	0.01 (0.01)	0.228

Contrast Sensitivity, mesopic [d]	-0.06 (0.03)	0.024 *	-0.02 (0.00)	<0.001 ***	-0.01 (0.00)	0.033 *	0.00 (0.01)	0.791
Contrast Sensitivity, contrast % [c]	0.08 (0.25)	0.740	0.09 (0.07)	0.168	0.10 (0.12)	0.404	0.09 (0.14)	0.537
Contrast Sensitivity, number of letters [c]	1.44 (2.93)	0.625	3.97 (1.98)	0.049	-0.51 (0.67)	0.457	-2.34 (2.69)	0.387

The p-values for baseline differences between high and low pfGCIPL subgroups were derived from the group effect in LMMs, using low pfGCIPL PD subgroup as reference. Separate models were adjusted for each subgroup to estimate β_{time} (annualized rate of change) and were adjusted for age at baseline and sex, and for cognitive scores also for education. β_{int} shows the adjusted estimated difference in the rate of change between pfGCIPL subgroups.

[a] Higher scores on the TMT indicate seconds to complete the task (i.e., worse performance).

Due to a protocol change, primary visual function was measured differently:

[b] Retro-illuminated cabinet at 4 meter using 100% contrast ETDRS and 2.5% contrast Sloan charts.

[c] Precision Vision Visual Acuity Test (PVVAT) digital software at 4m

[d] Pelli-Robson Test at 1m

Statistical significance: *p<0.05, ** p<0.01, *** p<0.001

Abbreviations: H&Y, Hoenh & Yahr scale; LEDD, levodopa-equivalent daily dose; MoCA, Montreal Cognitive Assessment; PD, Parkinson's disease; pfGCIPL, parafoveal ganglion cell-inner plexiform layer complex.

Supplementary Table 7. Baseline pRNFL thickness and annualized pRFNL thinning rate in PD patients depending on pfGCIPL subgroup

	β group (SE)	p	β int (SE)	p
pRNFL, mean	-7.77 (1.50)	<0.001	0.09 (0.16)	0.543
pRNFL, superior	-8.25 (1.75)	<0.001	0.07 (0.16)	0.664
pRNFL, inferior	-8.22 (2.40)	<0.001	0.14 (0.32)	0.474
pRNFL, nasal	-6.17 (2.28)	0.007	0.20 (0.23)	0.393
pRNFL, temporal	-8.58 (2.67)	0.002	0.10 (0.32)	0.734

$ß_{group}$ represents the estimated baseline difference coefficient of low pfGCIPL PD subgroup compared to high pfGCIPL PD subgroup, derived from age- and sex-adjusted LMMs. $ß_{interaction}$ indicates the difference in the annualized estimated thinning rate in low vs. high pfGCIPL PD subgroup. *Abbreviations*: PD, Parkinson's disease; pRNFL, peripapillary nerve fiber layer; SE, Standard Error.

Supplementary Table 8. Topcon OCT devices used in AlzEye study and the corresponding number of images used in the current dataset

**OCT device**	**No. of images**
3D OCT-1000, FD-OCT	361
3D OCT-1000, Mk2	96
3D OCT-2000	2516
DRI OCT Triton Plus	8

Abbreviations: OCT, Optical Coherence Tomography

Supplementary Methods

Participant Selection from AlzEye Database

The validation dataset used in this study was extracted from AlzEye, a retrospective cohort of 353157 subjects who attended Moorfields Eye Hospital NHS Foundation Trust (MEH) between January 2008 and April 2018 [1]. AlzEye includes retinal imaging, ophthalmic variables, and systemic disease labels defined by International Classification of Diseases (ICD) codes. The latter were obtained by linking MEH data with the National Health Service database of Hospital Episode Statistics (HES).

From the entire database, two groups were initially created with subjects who had macular OCT imaging ready to be analysed: PD (n=1283) and control (n=125110). PD was defined as having a HES with an ICD code for PD (G20). To create the final dataset, the process described in Figure 5 was followed.

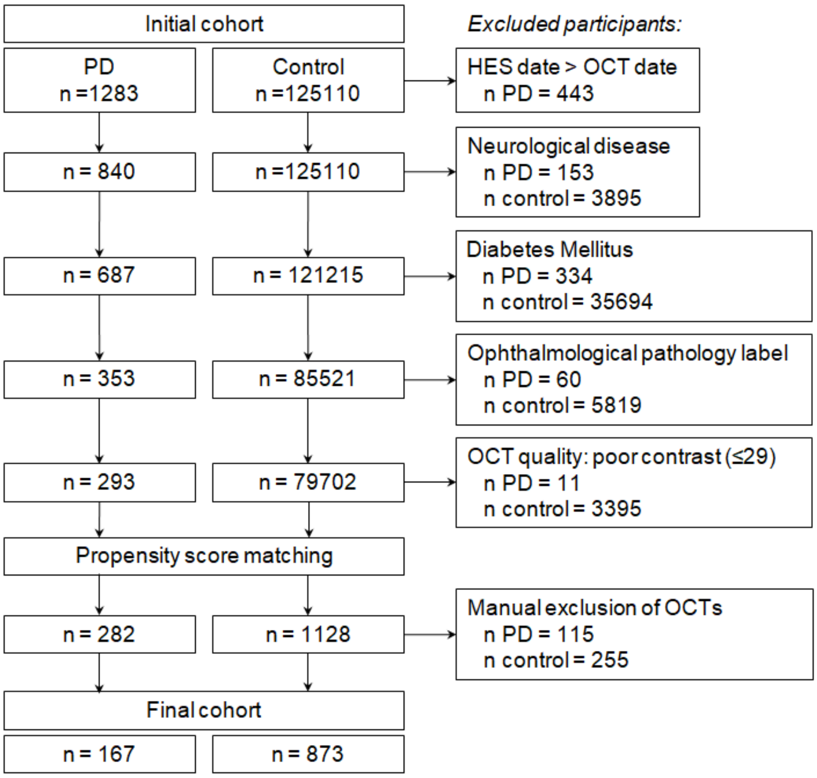

Supplementary Figure 5. Validation cohort flow chart. Participant and OCT data selection process from AlzEye database is shown.

First, all PD images before the first HES date with a PD code were removed to ensure that included PD images were acquired after disease onset. Second, we excluded individuals with ICD codes for the following neurological diseases: Alzheimer's disease, cerebral amyloid angiopathy, Huntington's disease, motor neuron disease, multiple sclerosis, progressive supranuclear palsy, schizophrenia, subarachnoid haemorrhage, and vascular dementia. Then, participants with Diabetes Mellitus were excluded. Additionally, eyes with ophthalmological labels for age-related macular degeneration, and glaucoma were excluded.

Next, OCT volumes with poor contrast were removed based on the image quality index provided by Topcon device metadata. The employed threshold (≦ 29) was determined by maximising the Youden Index on a separate dataset that included manually graded UK Biobank Topcon OCT images. Then, four controls were selected for each PD subject by means of propensity score matching based on age, sex, ethnicity, and hypertension status. In the last step, included OCT images were manually inspected by two experienced operators to remove the ones with poor quality and the presence of artefacts, such as motion artefacts, poor image centration, segmentation errors, and ocular lesions. The final cohort included 167 PD patients and 873 controls (sTable 2).

Reference Control Sample for OCT

Voluntary participants were prospectively enrolled between January 2019 and March 2023 at the Department of Ophthalmology of Cruces University Hospital to undergo retinal OCT. Inclusion criteria were to be older than 18 years and be able to collaborate in OCT acquisition. Exclusion criteria included: 1) history of severe smoking (>20 cigarettes/day); 2) heavy alcohol use (>4 drinks/day for men or >3 drinks/day for women), 3) diagnosis of any type or grade of diabetes mellitus, 4) diagnosis of uncontrolled or resistant elevated blood pressure, 5) obesity (body mass index > 30), 6) history of consumption of drugs or medications known to induce retinal toxicity or cognitive impairment, 7) chronic inflammatory systemic diseases (e.g., lupus erythematosus, sarcoid, Behçet's disease), 8) carotid or cerebral artery disease, 9) history of brain trauma or other structural brain lesions, 10) central nervous system diseases. Patients with well-controlled high blood pressure (hypertension) without complications were included in the study. We also excluded candidates with spherical equivalent refractive error > 4.00 diopters, > 3.00 diopters of astigmatism, or any other ocular condition potentially affecting OCT measures, following OSCAR-IB criteria [4].

To calculate the cut-off point of "low" pfGCIPL thickness value, 415 participants with a similar age range to test dataset (40 to 80 years old) were selected (sFig.6A). In this sample, 61.2%

were women. The pfGCIPL thickness distribution of the control reference group is shown in sFig.6B. The upper limit of the lower quartile was used as the cut-off point (89.8 µm) to divide participants into low and high pfGCIPL groups.

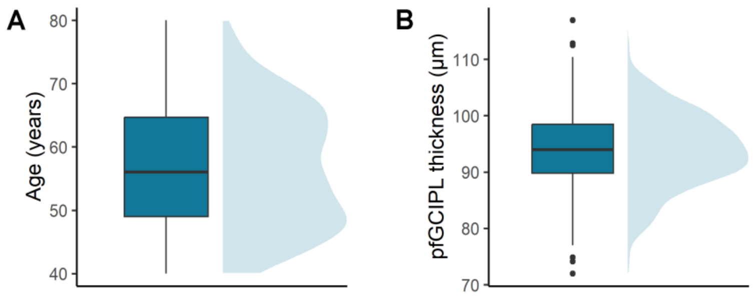

Supplementary Figure 6. Box plots and flat violin plots of age (A) and pfGCIPL (B) distribution in the reference control sample. *Abbreviations:* pfGCIPL, parafoveal ganglion cell-inner plexiform layer complex.
